# Association of arsenic-induced cardiovascular disease susceptibility with genetic polymorphisms

**DOI:** 10.1038/s41598-021-85780-8

**Published:** 2021-03-18

**Authors:** Mohammad Al-Forkan, Fahmida Binta Wali, Laila Khaleda, Md. Jibran Alam, Rahee Hasan Chowdhury, Amit Datta, Md. Zillur Rahman, Nazmul Hosain, Mohammad Fazle Maruf, Muhammad Abdul Quaium Chowdhury, N. K. M. Mirazul Hasan, Injamamul Ismail Shawon, Rubhana Raqib

**Affiliations:** 1 Department of Genetic Engineering and Biotechnology, Faculty of Biological Sciences, University of Chittagong, Chittagong, 4331 Bangladesh; 2 Department of Biochemistry and Biotechnology, University of Science and Technology, Chittagong (USTC), Foy’s Lake, Chittagong, 4202 Bangladesh; 3 Department of Pathology, Chittagong Medical College, Chittagong, 4203 Bangladesh; 4 Department of Cardiac Surgery, Chittagong Medical College Hospital, Chittagong, 4203 Bangladesh; 5 Infectious Disease Division, International Centre for Diarrhoeal Disease Research, Bangladesh (icddr,b), Mohakhali, Dhaka, 1212 Bangladesh

**Keywords:** Genetics, Molecular biology, Biomarkers, Medical research, Risk factors

## Abstract

Inorganic arsenic (iAs) exposure has been reported to have an impact on cardiovascular diseases (CVD). However, there is not much known about the cardiac tissue injury of CVD patients in relation to iAs exposure and potential role of single nucleotide polymorphisms (SNPs) of genes related to iAs metabolism, oxidative stress, endothelial dysfunction and inflammation which may play important roles in such CVD cases. In this dual center cross-sectional study, based on the exclusion and inclusion criteria, we have recruited 50 patients out of 270, who came from known arsenic-affected and- unaffected areas of mainly Chittagong, Dhaka and Rajshahi divisions of Bangladesh and underwent open-heart surgery at the selected centers during July 2017 to June 2018. We found that the patients from arsenic affected areas contained significantly higher average iAs concentrations in their urine (6.72 ± 0.54 ppb, *P* = 0.028), nail (529.29 ± 38.76 ppb, *P* < 0.05) and cardiac tissue (4.83 ± 0.50 ppb, *P* < 0.05) samples. Patients’ age, sex, BMI, hypertension and diabetes status adjusted analysis showed that patients from arsenic-affected areas had significantly higher iAs concentration in cardiac tissue (2.854, 95%CI 1.017–8.012, *P* = 0.046) reflecting higher cardiac tissue injury among them (1.831, 95%CI 1.032–3.249, *P* = 0.039), which in turn allowed the analysis to assume that the iAs exposure have played a vital role in patients’ disease condition. Adjusted analysis showed significant association between urinary iAs concentration with AA (*P* = 0.012) and AG (*P* = 0.034) genotypes and cardiac iAs concentration with AA (*P* = 0.017) genotype of *AS3MT* rs10748835. The AG genotype of *AS3MT* rs10748835 (13.333 95%CI 1.280–138.845, *P* = 0.013), AA genotype of *NOS3* rs3918181 (25.333 95%CI 2.065–310.757, *P* = 0.002), GG genotype of *ICAM1* rs281432 (12.000 95%CI 1.325–108.674, *P* = 0.010) and AA genotype of *SOD2* rs2758331 (13.333 95%CI 1.280–138.845, *P* = 0.013) were found significantly associated with CVD patients from arsenic-affected areas. Again, adjusted analysis showed significant association of AA genotype of *AS3MT* rs10748835 with CVD patients from arsenic affected areas. In comparison to the reference genotypes of the selected SNPs, AA of *AS3MT* 10748835, AG of *NOS3* rs3918181 and AC of rs3918188, GG of *ICAM1* rs281432, TT of *VCAM1* rs3176867, AA of *SOD2* rs2758331 and GT of *APOE* rs405509 significantly increased odds of cardiac tissue injury of CVD patients from arsenic affected areas. The results showed that the selected SNPs played a susceptibility role towards cardiac tissue iAs concentration and injury among CVD patients from iAs affected areas.

## Introduction

Arsenic contamination, more specifically the inorganic arsenic (iAs) contamination has been identified as a major health threat to the millions of people worldwide, including Bangldesh^1^. According to WHO, the acceptable limit of iAs in groundwater is 10 μg/L. However, Due to its geogenic origin, in Bangladesh, the iAs concentration in groundwater is often above 50 μg/L (national standard) and sometimes exceeds WHO allowed level at as much as 50 fold higher concentration^2, 3^. Human exposure to iAs has been found to be related with severe health consequences including several types of malignancies, e.g. skin, bladder, liver and lung; and various non-cancerous diseases including cardiovascular diseases (CVD)^4–6^, though heart is not the primary depository organ of iAs in human body. However, iAs exerts its toxicity on multiple organs in the human body and in our study, we focused on the heart, as evidence suggests that iAs has adverse toxic effects on this vital organ^7^.

Among the non-communicable diseases (NCDs including different types of cancers, diabetes, chronic respiratory diseases, CVD and others), CVD is the top ranked cause of death worldwide^8^. Recent studies have reported that chronic exposure to iAs (low to moderate level) is widely prevalent for the risk of CVD. However, several other important factors including age, gender and alterations in the iAs biotransformation and oxidative stress response genes can also influence the susceptibility towards iAs related diseases among individuals ^9–12^. The hypothesis that iAs induces oxidative stress and vascular inflammation, which play central role in developing atherosclerosis and CVD, has been supported by recent experimental studies^13, 14^. Moreover, polymorphisms of the genes responsible for iAs biotransformation (e.g. arsenic-3-methyltransferase, *AS3MT*), oxidative stress (e.g. nitric oxide synthase 3, *NOS3* and superoxide dismutase 2, *SOD2*), inflammation/endothelial dysfunction (e.g. intercellular adhesion molecule-1, *ICAM1* and soluble vascular adhesion molecule­1, *VCAM1*); and inflammation (e.g. apolipoprotein E, *APOE*), can be attributed as one of the reasons for the difference in individual susceptibility to iAs-induced CVD^11, 13, 15–18^. Some variants of these genes may alter the efficiency of iAs biotransformation and thus may cause difference in the level of susceptibilities of the cardiovascular effects of iAs exposure.

When iAs enters human body, pentavalent arsenate (AsV) is reduced to arsenite (AsIII) and subsequently converted to methylated forms (mono-, di- and tri-methylated forms) as a detoxification process catalyzed by arsenite methyl transferase enzyme (AS3MT)^19–21^. The gene *AS3MT* (10q24.32, ~ 32 kb) contains 11 exons which encodes the methyltransferase catalysing the biotransformation of trivalent inorganic arsenicals to their respective non-toxic derivatives^22–24^. It has been observed that within the same population, individual metabolic responses to different iAs metabolites vary which can be attributed to the polymorphism of *AS3MT* gene^25, 26^. Polymorphic variations in *AS3MT* gene may cause differences in the individual response in such iAs biotransformation process and thus may influence iAs-induced health effects. In addition, the pathophysiological factors including high oxidative stress along with recurrent low-grade inflammation are important for the development of CVD e.g. hypertension and atherosclerosis^27^. Multiple genetic polymorphisms *SOD2* and *APOE* are likely to be associated with the development of CVD as these genes are related with oxidative stress and chronic inflammation^28^. *NOS3* gene is involved in oxidative stress and two of its reference SNPs (rs3918181 A/G and rs3918188 A/C) have been marked for their association with CVD and coronary artery disease risk and ischemic stroke^28–31^. eNOS in humans produces NO from L-arginine and through this metabolic reaction, L-arginine regulates blood pressure and helps to control endothelium-dependent vasodilatation^30, 32, 33^. Some SNPs of *NOS3* gene can affect NOS3 activity, resulting in endothelial dysfunction and might play a vital role in atherosclerotic heart disease formation^30^. Maintenance of blood pressure homeostasis and vascular integrity are driven by the production of nitric oxide, which is catalysed by the enzyme *eNOS*. Variation in the expression of *eNOS* gene and its activity has been linked to primary hypertension. Moreover, a growing body of evidence has reported an association between primary hypertension and polymorphisms of the *eNOS* gene^32^. Additionally, the risk factors for atherosclerosis (e.g. hyperlipidemia, low-density lipoprotein and hypertension) are also found to be related with the level of adhesion molecules, i.e. *ICAM1* and *VCAM1*^34, 35^. Monocyte recruitment, which regulates the aggregation of atherosclerotic plaques, is facilitated by the endothelial adhesion molecules which include selectins, ICAM-1 and VCAM-1^28, 36, 37^.

Although genetic constitution responsible for the supposed protection or predilection to the effects of iAs exposure may play a critical role in CVD pathogenesis, to our knowledge, not much research has been done on this association in the Bangladesh perspective. Therefore, to investigate whether there is any association between iAs exposure and CVD incidence in Bangladesh perspective, we designed a cross-sectional study where 50 CVD patients were recruited from a total of 270 patients who underwent open-heart surgery at the Department of Cardiac Surgery, Chittagong Medical College Hospital, Chittagong and National Heart Foundation Hospital & Research Institute, Dhaka in between July 2017 to June 2018. The inclusion criteria include non-congenital CVD patients with the age range 20–70 years, willingness of the patients to participate in our study and adequacy of the samples (nail, urine and cardiac tissue). We excluded patients with congenital CVDs (see Supplementary Method 1). All the patients recruited in the study were pre-operatively diagnosed with blocks created by atherosclerotic plaques in the arteries including triple vessel diseases (TVD), double vessel diseases (DVD) and left main coronary artery (LMCA) diseases. Based on their residential area, patients were divided into two groups after inclusion in this study, whether or not they live in an arsenic contaminated area. The patients who came from areas with documented > 50 ppb arsenic in the groundwater were grouped as iAs-exposed whereas, iAs-unexposed group comprised with the patients who came from areas with < 10 ppb arsenic in the groundwater. Arsenic level in the ground water of patients’ residential area were determined based on the previously published articles^38–45^. Furthermore, we measured the arsenic exposure of the patients by measuring the iAs concentration in their urine and nail samples. Our recruited patients were mostly from the well documented arsenic contaminated areas of Chittagong, Dhaka and Rajshahi divisions of Bangladesh (see Supplementary Dataset).

We measured the cardiac tissue iAs concentration of the patients and scored cardiac tissue injury by histopathology. We further evaluated the genetic susceptibility of iAs exposure and subsequent CVD risk, i.e. if the cardiovascular effects of iAs exposure differs due to the polymorphic variations in genes responsible for iAs methylation (*AS3MT* rs10748835), oxidative stress (*NOS3* rs3918181, rs3918188; and *SOD2* rs2758331) and inflammation/endothelial dysfunction (*ICAM1* rs281432 and *VCAM1* rs3176867) within CVD patients. Most of the selected SNPs are within the intronic region of the genes. Unlike the exonic variants, intronic SNPs exhibit difference in association among populations. We wanted to explicate the associations of a selected list of SNPs of intronic origin that were previously found strongly associated with CVD and iAs metabolism in some populations. The intrinsic function of these SNPs are slowly being understood, therefore, the association of these intronic SNPs with diseases in different populations needs to be clarified so that further studies can emphasize on the functional aspect of their disease association. In short, our study aimed to check the existence of any association between single nucleotide polymorphisms (SNPs) of the genes mentioned above and CVD in the patients who were living in the aforementioned areas of Bangladesh and were exposed to varied ranges of iAs.

## Results

### Patient characteristics and iAs exposure analysis

Table 1 shows the general characteristics of study subjects. With regard to gender, age, smoking and BMI, there were no significant differences among the subjects who came from iAs-affected areas and the subjects who came from iAs-unaffected areas. iAs-exposed subjects had higher iAs concentrations (*P* < 0.05) in urine, nail and cardiac tissues than the iAs-unexposed subject group. iAs-exposed subjects also had significantly more cases hypertension (*P* < 0.05) than the iAs-unexposed subjects. In Table 2, the iAs exposure measurement of the patients was done. Patients’ age, sex, BMI, hypertension and diabetes status adjusted binary logistic regression showed that patients from iAs-affected areas had higher odds of having urine (1.221, 95%CI 0.868–1.716), nail (2.084, 95%CI 0–3.547E + 34) and cardiac (2.854, 95%CI 1.017–8.012) total iAs concentrations. The cardiac iAs concentration of the patients from iAs-affected areas showed significant value in this regard (*P* = 0.046).

**Table 1:** Distribution of study population characteristics (n = 50). The *P* values in ‘bold’ are significant. The significance level is *P* < 0.05. *Age and BMI were shown as mean ± SE and analyzed by Student’s *t* test. **Sex, habit of smoking, Hypertension and Diabetes Mellitus (DM) were shown as percent and analyzed by Chi-square test. ^#^The values of iAs concentrations were shown as mean ± SE and analyzed by Student’s *t* test.

Variables	Patients from iAs-affected areas (n = 36)	Patients from iAs-unaffected areas (n = 14)	X^2^	*P* value
Age (years)	48.97 ± 1.71	49.64 ± 1.44		0.766*
**Sex**				
Male (%)	28 (77.78%)	9 (64.29%)	0.954	0.474**
Female (%)	8 (22.22%)	5 (35.71%)		
**Habit of smoking**				
Smoker (%)	24 (66.67%)	8 (57.14%)	0.397	0.533**
Non-smoker (%)	12 (33.33%)	6 (42.86%)		
Body mass index (BMI) (mean ± SE), (Kg/m^2^)	23.88 ± 0.60	22.99 ± 1.10		0.491*
**Hypertension**				
Yes	22 (61.11%)	2 (14.29%)	8.855	0.004**
No	14 (38.89%)	12 (85.71%)		
Diabetes mellitus (DM)				
Yes	16 (44.44%)	8 (57.14%)	0.651	0.533**
No	20 (55.56%)	6 (42.86%)		
**Total inorganic arsenic concentrations (ppb)**^#^				
Cardiac iAs conc. (ppb)	4.83 ± 0.50	2.11 ± 0.24		** < 0.05**
Urinary iAs conc. (ppb)	6.72 ± 0.54	4.63 ± 0.73		**0.028**
Nail iAs conc. (ppb)	529.29 ± 38.76	197.19 ± 12.17		** < 0.05**

**Table 2:** Total inorganic arsenic exposure measurement of the patients. Here, binary logistic regression was done for the association between patients’ residence with the measured arsenic in cardiac tissue, urine and nail by adjusting the analysis for age, sex, BMI, hypertension and diabetes status of the patients. Significant values (*P* < 0.05) are typed in bold font.

Variables	Patients from iAs-affected areas (n = 36) Mean ± SE	Patients from iAs-unaffected areas (n = 14) Mean ± SE	Odds ratio (95% CI)	*P* value
**Total inorganic arsenic concentrations (ppb)**				
Cardiac iAs conc. (ppb)	4.83 ± 0.50	2.11 ± 0.24	2.854 (1.017–8.012)	**0.046**
Urinary iAs conc. (ppb)	6.72 ± 0.54	4.63 ± 0.73	1.221 (0.868–1.716)	0.251
Nail iAs conc. (ppb)	529.29 ± 38.76	197.19 ± 12.17	2.084 (0–3.547E + 34)	0.985
Cardiac tissue injury score	6.17 ± 0.28	3.64 ± 0.58	1.831 (1.032–3.249)	**0.039**

### Genotypic and allelic frequencies

Adjusted binary logistic regression revealed that the AA (*P* = 0.012) and AG (*P* = 0.034) genotypes of rs10748835 of *AS3MT* were significantly associated with urinary iAs concentration (Table 3). The presence of AA and AG genotypes was found to increase the odds of urinary iAs concentration (1.917, 95%CI 0.965–3.806). Only the AA (*P* = 0.017) genotype was significantly associated with cardiac tissue iAs concentration. This genotype also increased the odds of cardiac iAs concentration (2.988, 95%CI 0.669–13.338).

**Table 3:** Association between *AS3MT* rs10748835 polymorphism and total inorganic arsenic concentration in urine and cardiac tissues of patients. Here, binary logistic regression was done by adjusting the association with patients’ age, sex, BMI, hypertension and diabetes status. There was no association between the *AS3MT* polymorphism and nail arsenic concentration (data not shown). The significant values (*P* < 0.05) are typed in bold font.

Gene	SNP	Genotype	iAs conc. (Mean ± SE)	Odds ratio (95%CI)	*P* value
**Urinary iAs conc. (ppb)**					
***AS3MT***	rs10748835	AA	4.73 ± 0.67	Reference	**0.012**
		AG	5.21 ± 0.45	94.266 (1.414–6285.781)	**0.034**
		GG	7.55 ± 0.83	0.103 (0.003–3.537)	0.208
		*OR of Urinary iAs = 1.917 (0.965–3.806)*			
**Cardiac iAs conc. (ppb)**					
***AS3MT***	rs10748835	AA	1.94 ± 0.22	Reference	**0.017**
		AG	4.09 ± 0.60	13.556 (0.481–382.223)	0.126
		GG	5.11 ± 0.69	0.126 (0.005–3.419)	0.219
		*OR of Cardiac tissue iAs = 2.988 (0.669–13.338)*			

The genotypic and allelic frequencies of the seven SNPs of *AS3MT, NOS3, ICAM1, VCAM1*, *SOD2* and *APOE* genes were analyzed by Chi-square test (Table 4 and Supplementary Table S1). Higher frequencies were found for AG genotype of *AS3MT* rs10748835 (13.333 95%CI 1.280–138.845, *P* = 0.013), AA genotype of *NOS3* rs3918181 (25.333 95%CI 2.065–310.757, *P* = 0.002), GG genotype of *ICAM1* rs281432 (12.000 95%CI 1.325–108.674, *P* = 0.010) and AA genotype of *SOD2* rs2758331 (13.333 95%CI 1.280–138.845, *P* = 0.013) in iAs-exposed CVD patients group. No association was found among iAs-exposed and unexposed groups for the distribution of the other three selected SNPs. Furthermore, we found significant differences (*P* < 0.05) in the distribution of A allele of *NOS3* rs3918181; G allele of *ICAM1* rs281432 and A allele of *SOD2* rs2758331genes among iAs-exposed and unexposed patient’s groups (Supplementary Table S1). Adjusted binary logistic regression showed that AA of *AS3MT* 10748835, AG of *NOS3* rs3918181 and AC of rs3918188, GG of *ICAM1* rs281432, TT of *VCAM1* rs3176867, AA of *SOD2* rs2758331 and GT of *APOE* rs405509 significantly increased odds of cardiac tissue injury of CVD patients from arsenic affected areas. Moreover, the adjusted logistic regression analysis revealed that the AA genotype of *AS3MT* rs10748835 was significantly associated with the patients from iAs-affected areas (Table 4).

**Table 4:** Distribution of genotype frequencies among the patient groups. The data were analyzed by Chi-square Test and shown as mean ± SE value. The association analyses were then adjusted for age, sex, BMI, hypertension and diabetes and analyzed by binary logistic regression. Significant values (*P* < 0.05) are typed in bold font.

Gene	SNP	Genotype	Number (%) of patients from iAs-affected areas (n = 36)	Number (%) of patients from iAs-unaffected areas (n = 14)	OR(95%CI) *P* value	Adjusted OR(95%CI) *P* value	Adjusted OR(95%CI) of Cardiac Injury *P* value
***AS3MT***	rs10748835	AA	6 (16.67%)	5 (35.71%)	Reference	**0.027** (Reference)	1.950 (1.044–3.643) ***P = 0.036***
		AG	16 (44.44%)	1 (7.14%)	13.333 (1.280–138.845) **0.013**	11.276 (0.260–488.335) 0.208	–
		GG	14 (38.89%)	8 (57.14%)	1.458 (0.335–6.347) 0.614	0.172 (0.005–6.320) 0.338	–
***NOS3***	rs3918181	GG	3 (8.33%)	4 (28.57%)	Reference	0.220 (Reference)	**–**
		AG	14 (38.89%)	9 (64.29%)	2.074 (0.373–11.528) 0.400	13.027 (0.485–350.152) 0.126	2.201 (1.167–4.153) ***P = 0.015***
		AA	19 (52.78%)	1 (7.14%)	25.333 (2.065–310.757) **0.002**	1.197 (0.087–16.378) 0.893	–
	rs3918188	CC	21 (58.33%)	6 (42.86%)	Reference	0.176 Reference	**–**
		AC	9 (25%)	6 (42.86%)	0.429 (0.108–1.695) 0.222	1.862 (0.055–63.595) 0.730	2.567 (1.317–5.003) ***P = 0.006***
		AA	6 (16.67%)	2 (14.29%)	0.857 (0.136–5.395) 0.869	0.114 (0.011–1.231) 0.074	–
***ICAM1***	rs281432	CC	12 (33.33%)	8 (57.14%)	Reference	0.157 (Reference)	**–**
		GG	18 (50%)	1 (7.14%)	12.000 (1.325–108.674) **0.010**	18.772 (0.858–410.892) 0.063	2.358 (1.238–4.493) ***P = 0.009***
		CG	6 (16.67%)	5 (35.71%)	0.800 (0.181–3.536) 0.768	1.133 (0.122–10.501) 0.912	–
***VCAM1***	rs3176867	CC	19 (52.78%)	7 (50%)	Reference	0.451 (Reference)	**–**
		TT	11 (30.56%)	3 (21.43%)	1.351 (0.289–6.320) 0.702	46.998 (0.077–28,730.920) 0.240	3.520 (1.371–9.036) ***P = 0.009***
		AA	4 (11.11%)	3 (21.43%)	0.491 (0.087–2.770) 0.416	–	–
		CT	2 (5.56%)	1 (7.14%)	0.737 (0.057–9.457) 0.814	5.047 (0.147–172.826) 0.369	–
***SOD2***	rs2758331	CC	6 (16.67%)	5 (35.71%)	Reference	0.148 (Reference)	**–**
		AC	14 (38.89%)	8 (57.14%)	1.458 (0.335–6.347) 0.614	0.714 (0.054–9.463) 0.798	–
		AA	16 (44.44%)	1 (7.14%)	13.333 (1.280–138.845) **0.013**	23.674 (0.642–872.562) 0.086	2.490 (1.187–5.221) ***P = 0.016***
***APOE***	rs405509	GG	6 (16.67%)	5 (35.71%)	Reference	0.337 (Reference)	–
		GT	18 (50%)	6 (42.86%)	2.500 (0.556–11.250) 0.226	6.060 (0.548–67.077) 0.142	2.337 (1.225–4.459) ***P = 0.010***
		TT	12 (33.33%)	3 (21.43%)	3.333 (0.588–18.891) 0.165	3.138 (0.244–40.432) 0.381	–

### Difference in SNP genotype distribution with total iAs concentration in urine, nail and cardiac tissue

The difference of genetic polymorphisms with measured iAs concentrations in biological samples (urine, nail and cardiac tissue) were analyzed by Student’s *t* test (Table 5). In case of urinary iAs concentration, the SNP genotypes of *AS3MT* rs10748835 (*P* = 0.047), *NOS3* rs3918181 (*P* < 0.005), *ICAM1* rs281432 (*P* = 0.003) and *VCAM1* rs3176867 (*P* = 0.035) differed significantly among the patients. The genotypes of *NOS3* rs3918181 (*P* = 0.001) and *SOD2* rs2758331 (*P* = 0.011) were significantly different among the patients in case of nail iAs concentration. Finally, *AS3MT* rs10748835 (*P* < 0.005) and *NOS3* rs3918181 (*P* = 0.0001) genotypes differed significantly among the patients in case of cardiac tissue iAs concentration. The other SNP genotypes did not show any significant differences for iAs concentration in urine, nail and cardiac tissue samples of the patients. The significant distribution differences in the genotypes of the aforementioned selected SNPs among the patients for iAs concentration in the samples reflect their possible susceptibility role towards iAs exposure.

**Table 5:** Association of genetic polymorphism with total iAs concentration in urine, nail and cardiac tissue. The data were analyzed by Student’s *t* test and shown as mean ± SE value. Significant values (*P* < 0.05) are typed in bold font.

Gene	SNP	Genotype	Urinary iAs conc. (Mean ± SE)	Nail iAs conc. (Mean ± SE)	Cardiac iAs conc. (Mean ± SE)
*AS3MT*	rs10748835	AA	4.73 ± 0.67	337.95 ± 48.54	1.94 ± 0.22
		AG + GG	6.53 ± 0.53	464.04 ± 42.17	4.67 ± 0.47
		*P* value	**0.047**	0.06	** < 0.005**
*NOS3*	rs3918181	GG	3.58 ± 0.38	235.31 ± 43.34	1.87 ± 0.36
		AG + AA	6.54 ± 0.49	466.08 ± 38.51	4.42 ± 0.44
		*P* value	** < 0.005**	**0.001**	**0.0001**
	rs3918188	CC	6.29 ± 0.69	461.85 ± 51.21	4.76 ± 0.00
		AC + AA	5.94 ± 0.57	470.94 ± 55.71	4.09 ± 0.52
		*P* value	0.688	0.904	0.427
*ICAM1*	rs281432	CC	4.68 ± 0.44	377.59 ± 53.02	3.21 ± 0.57
		CG + GG	7.09 ± 0.64	475.44 ± 46.09	4.64 ± 0.54
		*P* value	**0.003**	0.171	0.075
*APOE*	rs405509	GG	7.32 ± 1.21	416.23 ± 79.77	2.93 ± 0.56
		GT + TT	6.51 ± 0.67	441.96 ± 39.61	3.76 ± 0.44
		*P* value	0.566	0.777	0.248
*SOD2*	rs2758331	CC	7.86 ± 1.11	305.98 ± 51.72	3.39 ± 0.80
		AC + AA	5.64 ± 0.47	492.88 ± 45.17	4.26 ± 0.46
		*P* value	0.087	**0.011**	0.367
*VCAM1*	rs3176867	CC	6.77 ± 0.62	469.79 ± 53.57	4.79 ± 0.56
		CT + TT	5.00 ± 0.52	422.36 ± 29.92	4.21 ± 0.66
		*P* value	**0.035**	0.444	0.513

### Association between patients’ characteristics and genetic polymorphisms

The association between demographic risk factors of patients and the polymorphisms of the genes *AS3MT, NOS3, ICAM1, VCAM1*, *SOD2* and *APOE* were analyzed. No significant differences were found (Supplementary Table S2). Age and BMI of the subjects having different genotypes of these SNPs, were also not found to be significantly different by ANOVA analysis. Among the study population, the majority (37/50) were male and 17 patients had no history of smoking including all the females, therefore, smoking habit was excluded from analysis adjustment in the binary logistic regression analysis.

### Association between clinical biomarkers of CVD and genetic polymorphism

**Figure 1:**
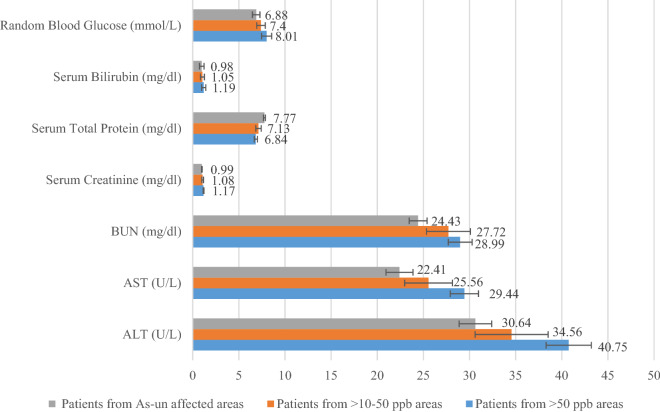
Levels of different biochemical parameters in different patient categories. Significant increase was observed for serum creatinine, BUN, ALT and AST among the patients from iAs-affected areas. Here, ALT = serum alanine amino-transferase, AST = serum aspartate amino-transferase, BUN = blood urea nitrogen. *P < 0.05, Student’s *t* test for Patient’s group from > 50 ppb iAs-affected areas vs iAs-unaffected areas.

We analyzed relevant biochemical parameters, the level of which may show significant fluctuation among the highly iAs-exposed and un-exposed groups including serum creatinine, serum total protein, blood urea nitrogen (BUN), serum bilirubin, aspartate aminotransferase (AST), alanine aminotransferase (ALT), and random blood glucose (RBS). We found that the level of Serum Creatinine, BUN, AST and ALT increased significantly among the patients from highly iAs-affected areas. The level of serum total protein was found to be significantly decrease among the patients from highly iAs-affected areas (Fig. 1; Supplementary Table S3).

**Figure 2:**
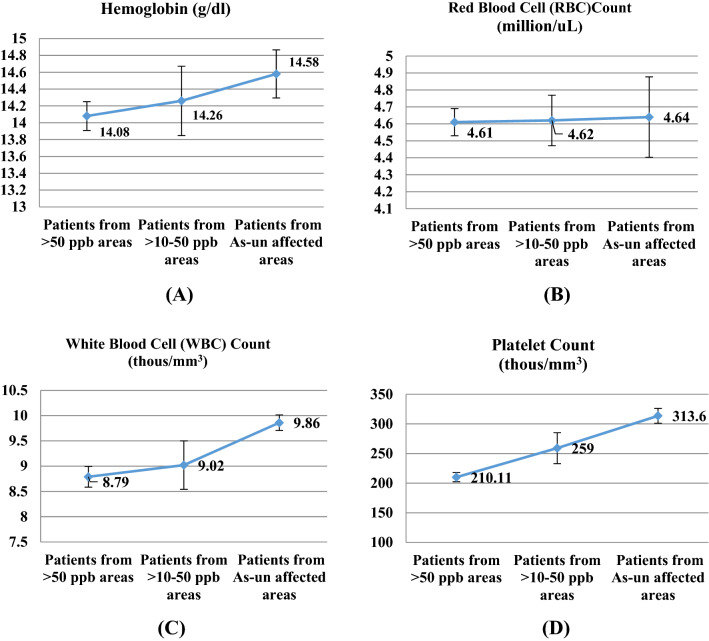
Levels of different biochemical parameters (**A**) hemoglobin, (**B**) red blood cell count, (**C**) white blood cell count and (**D**) platelet count in different patient categories. A decrease was seen in the patients exposed to higher concentrations of iAs (> 50 ppb).

According to our statistical analysis, there was a significant association (*P* < 0.05) between ALT levels and polymorphisms of *AS3MT* rs10748835, *NOS3* rs3918181 and rs3918188, AST levels were found to be significantly (*P* < 0.05) increased in patients with AA genotype of *NOS3* rs3918181 (Table 6). Patients with AA genotypes (*P* < 0.05) of *NOS3* rs3918181 and rs3918188 were also shown higher AST levels. The polymorphisms of *APOE* rs405509 also marked significant (*P* < 0.05) association with AST levels. BUN levels were found significantly associated with only *VCAM1* rs3176867, while no association were found with other genes. No significant difference was found in serum total protein levels with SNPs of our selected genes. For serum creatinine levels significant association (*P* < 0.05) was found with *AS3MT* rs10748835 (AA genotype), *APOE* rs405509 (GG genotype) and *SOD2* rs2758331 (AC genotype). Serum Bilirubin was only found associated (*P* < 0.05) with *NOS3* rs3918181 (GG genotype). RBS levels showed significant difference for the polymorphism of *APOE* rs405509 (AA genotype) (Table 6). iAs-exposed subjects showed lower average haemoglobin (Hb) level and red blood cell (RBC) count; however, white blood cell (WBC) and platelet counts were significantly lower than the iAs-unexposed patients (Fig. 2; Supplementary Table S4).

**Table 6:** Relationship between genetic polymorphisms and biochemical parameters. *AS3MT* rs10748835 A/G, *NOS3* rs3918181 A/G, *NOS3* rs3918188 A/C, *ICAM1* rs281432 C/G, *VCAM1* rs3176867 A/C/T (the data for AA genotype was excluded here), *SOD2* rs2758331 A/C and *APOE* rs405509 G/T. ALT = serum alanine amino-transferase, AST = serum aspartate amino-transferase, BUN = blood urea nitrogen. The data were analyzed by ANOVA. Significant values (*P* < 0.05) are typed in bold font.

Variables	Gene	SNPs loci	Genotype			*P* value
			AA	Aa	Aa	
ALT, U/L	*AS3MT*	rs10748835	**44 ± 4.29**	36.24 ± 2.52	33.36 ± 2.12	**0.045**
	*NOS3*	rs3918181	**41.5 ± 2.84**	35.95 ± 2.18	27.57 ± 2.65	**0.022**
		rs3918188	**46.88 ± 5.73**	36.07 ± 2.46	34.00 ± 1.94	**0.020**
	*ICAM1*	rs281432	39.5 ± 3.31	35.47 ± 2.11	33.64 ± 2.30	0.359
	*APOE*	rs405509	34.55 ± 4.21	39.46 ± 2.33	33.80 ± 2.98	0.277
	*SOD2*	rs2758331	33.41 ± 2.18	39.64 ± 2.98	35.82 ± 2.99	0.255
	*VCAM1*	rs3176867	38.08 ± 2.37	33.57 ± 2.19	33.67 ± 5.55	0.661
AST, U/L	*AS3MT*	rs10748835	30.91 ± 2.35	25.82 ± 1.52	26.18 ± 1.68	0.161
	*NOS3*	rs3918181	**29.80 ± 1.62**	24.04 ± 1.38	27.86 ± 3.69	**0.043**
		rs3918188	**35.00 ± 2.27**	24.96 ± 1.06	26.00 ± 2.37	**0.003**
	*ICAM1*	rs281432	29.30 ± 1.75	23.27 ± 1.74	26.42 ± 1.82	0.105
	*APOE*	rs405509	27.13 ± 1.57	21.45 ± 1.64	**30.47 ± 1.81**	**0.009**
	*SOD2*	rs2758331	27.12 ± 1.77	25.82 ± 1.78	28.64 ± 2.14	0.613
	*VCAM1*	rs3176867	26.35 ± 1.53	31.33 ± 4.70	29.73 ± 1.93	0.066
BUN, Mg/dl	*AS3MT*	rs10748835	26.15 ± 2.83	28.59 ± 0.61	26.89 ± 1.40	0.566
	*NOS3*	rs3918181	28.14 ± 1.61	27.02 ± 1.32	29.71 ± 2.39	0.629
		rs3918188	25.59 ± 2.68	27.50 ± 1.52	28.50 ± 1.33	0.559
	*ICAM1*	rs281432	28.14 ± 1.84	29.32 ± 2.12	27.26 ± 1.34	0.749
	*APOE*	rs405509	26.52 ± 2.07	27.65 ± 1.28	28.73 ± 1.95	0.713
	*SOD2*	rs2758331	27.59 ± 1.40	28.99 ± 1.41	25.41 ± 1.41	0.357
	*VCAM1*	rs3176867	27.93 ± 2.06	25.33 ± 2.19	**30.64 ± 2.06**	**0.042**
Serum Creatinine, mg/dl	*AS3MT*	rs10748835	**1.27 ± 0.08**	1.08 ± 4.69	1.03 ± 0.05	**0.004**
	*NOS3*	rs3918181	1.18 ± 0.05	1.06 ± 0.04	1.04 ± 0.04	0.103
		rs3918188	1.13 ± 0.06	1.01 ± 0.04	1.15 ± 0.04	0.091
	*ICAM1*	rs281432	1.17 ± 0.05	1.06 ± 0.06	1.07 ± 0.04	0.239
	*APOE*	rs405509	**1.25 ± 0.07**	1.05 ± 0.03	1.09 ± 0.05	**0.017**
	*SOD2*	rs2758331	1.04 ± 0.04	**1.2 ± 0.05**	1.02 ± 0.04	**0.011**
	*VCAM1*	rs3176867	1.07 ± 0.04	1.27 ± 0.15	1.2 ± 0.06	0.089
Serum Total Protein, mg/dl	*AS3MT*	rs10748835	6.94 ± 0.39	7.35 ± 0.15	7.11 ± 0.17	0.457
	*NOS3*	rs3918181	6.93 ± 0.21	7.23 ± 0.16	7.41 ± 0.34	0.343
		rs3918188	7.08 ± 0.45	7.28 ± 0.25	7.10 ± 0.13	0.794
	*ICAM1*	rs281432	6.98 ± 0.17	7.10 ± 0.27	7.37 ± 0.21	0.362
	*APOE*	rs405509	7.28 ± 0.29	6.99 ± 0.17	7.31 ± 0.22	0.478
	*SOD2*	rs2758331	7.38 ± 0.20	7.09 ± 0.18	6.91 ± 0.27	0.344
	*VCAM1*	rs3176867	7.16 ± 0.32	7.13 ± 0.52	7.41 ± 0.19	0.612
Serum Bilirubin, mg/dl	*AS3MT*	rs10748835	0.83 ± 0.09	0.79 ± 0.11	1.20 ± 0.20	0.121
	*NOS3*	rs3918181	0.94 ± 0.12	0.79 ± 0.08	**1.81 ± 0.52**	**0.003**
		rs3918188	0.92 ± 0.10	0.92 ± 0.17	1.05 ± 0.17	0.817
	*ICAM1*	rs281432	1.03 ± 0.15	0.73 ± 0.09	1.10 ± 0.22	0.389
	*APOE*	rs405509	0.79 ± 0.08	1.09 ± 0.18	0.97 ± 0.18	0.529
	*SOD2*	rs2758331	0.99 ± 0.12	1.05 ± 0.19	0.87 ± 0.19	0.800
	*VCAM1*	rs3176867	1.04 ± 0.16	0.70 ± 0.25	0.99 ± 0.18	0.900
RBS, mmol/l	*AS3MT*	rs10748835	7.33 ± 0.32	8.48 ± 0.84	7.03 ± 0.29	0.144
	*NOS3*	rs3918181	7.58 ± 0.55	7.63 ± 0.51	7.46 ± 0.64	0.986
		rs3918188	6.73 ± 0.23	8.35 ± 0.71	7.42 ± 0.45	0.247
	*ICAM1*	rs281432	7.61 ± 0.59	8.61 ± 0.90	6.97 ± 0.25	0.179
	*APOE*	rs405509	**9.22 ± 1.14**	7.18 ± 0.30	7.04 ± 0.41	**0.027**
	*SOD2*	rs2758331	6.84 ± 0.36	8.32 ± 0.64	7.27 ± 0.45	0.128
	*VCAM1*	rs3176867	7.92 ± 0.56	7.10 ± 1.00	7.26 ± 0.47	0.777

### Histopathology of cardiac tissue

**Figure 3:**
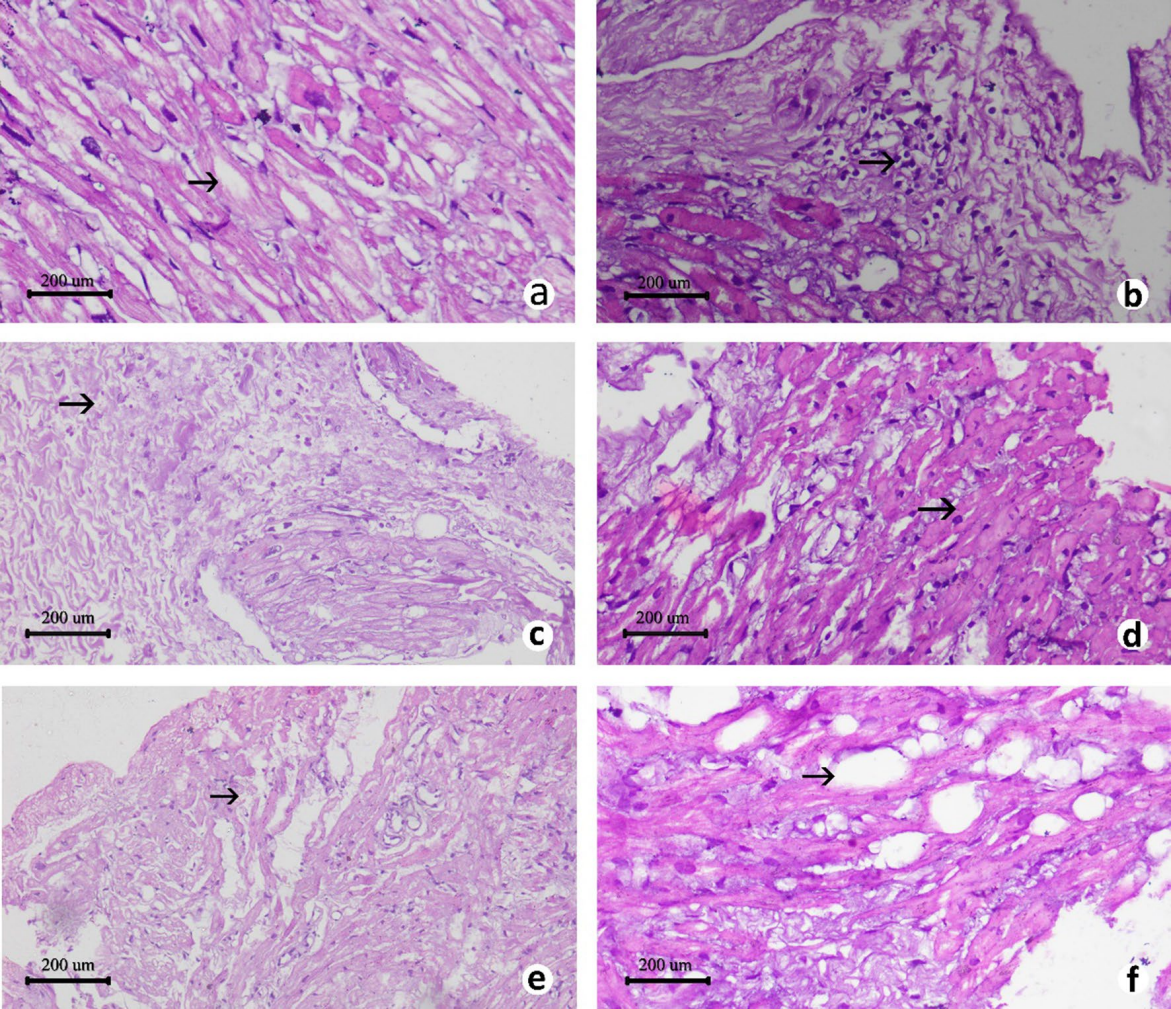
Histopathology images of cardiac tissue of patients. Representative photomicrographs (H and E stain) of cardiac tissue histopathology of patients showing different parameters; (**a**) edema (40x); (**b**) leukocyte infiltration (40x); (**c**) fibrosis (10x); (**d**) myocardial fiber swelling (40x); (**e**) fiber separation (10x); (**f**) fatty change (40x). The default scale bar for each micrograph was set at 200 μm.

Histopathological analysis revealed higher average cardiac tissue injuries in the patients from iAs affected areas. Although tissue injuries such as oedema, leukocyte infiltration, fibrosis, myocardial fibre swelling, fibre separation and fatty changes, were observed almost in all the patients, the significantly higher cardiac tissue injury scores (*P* < 0.05) were observed in the patients from iAs-affected areas than the patients from iAs-unaffected areas (Fig. 3; Supplementary Table S5). In iAs-exposed patients, the average cardiac tissue injury score was 5.64 ± 0.39. On the other hand, in the iAs-unexposed patients, the average cardiac tissue injury score was 3.64 ± 0.58. Notably, the average cardiac tissue injury score was 6.63 ± 0.42 in patients from > 50 ppb iAs affected areas and 4.78 ± 0.56 in patients from > 10–50 ppb iAs areas (Supplementary Table S5). Moreover, it was also found by the adjusted analysis that cardiac iAs concentration of a patient significantly increased the likelihood of cardiac tissue injury (OR = 1.831, 95%CI 1.032–3.249, *P* = 0.039) (Table 2). For this reason, our assumption was that the iAs deposition in the cardiac tissue of the patients along with the genotypic variations of the selected SNPs may have played an important role in the development of corresponding CVDs.

## Discussion

Recent epidemiological studies have confirmed that among the people living in the same area showed different susceptibility towards iAs exposure due to their genetic variation and such genetic differences alter individual susceptibilities to develop certain diseases, including cardiovascular diseases^12, 46^. It is acknowledged by multiple government and non-government organizations that a large portion of Bangladeshi residents are exposed to iAs on a daily basis ranging from low to higher concentration. However, among these exposed residents, a fraction, due to their genetic differences, may develop CVD as an effect of chronic iAs exposure^22, 47^. In this cross-sectional study, we reported that patients with CVD from iAs affected areas, found to be associated with higher iAs deposition in the nail, cardiac tissue and lower iAs-methylation capacity, which were indicated by high iAs concentration in the urine. The result also showed that polymorphisms in *AS3MT* SNP rs10748835, *NOS3* SNP rs3918181, *ICAM1* SNP rs281432 and *SOD2* SNP rs2758331, in concert with arsenic exposure increases the risk to CVD.

### AS3MT

Polymorphic alterations of the reference alleles of this gene are reported to cause reduction in the iAs metabolism efficiency, and in addition, A/G polymorphism at rs10748835 of *AS3MT* gene has been reported to be associated with iAs exposure and CVD ^48–50^. In their study, Gong and O’Bryant reported that patients with AG genotype at rs10748835 were at elevated risk of coronary heart diseases and hyperlipidemia than patients with AA genotype at the same SNP position^49^. Inconsistent with their findings, our results showed a significant difference in the distribution of AG genotype (13.333 95%CI 1.280–138.845, *P* = 0.013) between iAs-exposed and un-exposed patient groups (Table 4). We have also found significant differences in genotype distribution of *AS3MT* rs10748835 in urine (*P* = 0.047) and cardiac tissue (*P* < 0.005) iAs concentration among the patients (Table 5). Fu et al*.* investigated for the association between *AS3MT* rs10748835 polymorphism and iAs concentration in the urine after subsequent metabolism, where they found no association for this reference SNP. On the other hand, Engström et al*.* reported GG genotype significantly associated with urinary arsenic metabolism, meaning, people having GG genotype may have less efficient AS3MT enzyme than the people having AA genotype^24, 51^. We further adjusted our analysis by patients age, sex, BMI, hypertension and diabetes status in binary logistic regression for association analysis (Table 3). In this analysis also, the AG genotype of *AS3MT* rs10748835 showed significant association with increased urinary iAs concentration (94.266 95%CI 1.414–6285.781, *P* = 0.034). The AA genotype showed significant association with both urinary and cardiac iAs concentration (*P* = 0.012 and 0.017 respectively). Some other SNP association studies found no relations to disease susceptibility with this intronic variant, rs10748835 of *AS3MT* gene in disease susceptibility, however, the outcomes can vary possibly due to differences in populations and races^51, 52^. Therefore, the findings of our study allow us to suspect that AA and AG genotypes, specifically, the A allele might be the susceptibility factors for iAs exposure, cardiac iAs concentration and subsequent cardiac tissue injury and CVD in the Bangladeshi population.

### NOS3

The association of *NOS3* polymorphism and arsenic exposed CVD patients is controversial; one of the reasons might be the differences in populations and races. Among the Chinese Han population, Du et al*.* (2008) reported GG genotype and G allele of *NOS3* rs3918181 (A/G) to be strongly associated with ischemic stroke (male patients); whereas, Yang et al*.* (2014) showed no association of this SNP with primary hypertension^31, 32^. In our study subjects, we found AA genotype and A allele of *NOS3* rs3918181 (A/G) to be significantly associated with the CVD patients from iAs-affected area (Table 4). Moreover, the genotypic distribution of this SNP differed significantly among patients in regards to urine (*P* < 0.005), nail (*P* = 0.001) and cardiac tissue (*P* = 0.0001) iAs concentrations (Table 5). Garme et al*.* (2017) reported that the AA genotype of rs3918188 SNP of *NOS3* gene, may cause an individual to be more susceptible to develop T2DM^53^. Zhao et al*.* (2016) found no affirmative association between *NOS3* rs3918188 with CAD^30^. Consistently, no significant association was found with a specific genotype of *NOS3* rs3918188 with iAs-exposed CVD patients in this study (Table 4 and 5). It is evident that toenail iAs concentration reflects chronic exposure of iAs upto 6–12 months^54^. Higher concentrations of arsenic in the biological samples (nail and cardiac tissue) of the patients indicate the chronic exposure of them to arsenic. The likelihood of cardiac injury due to the presence of AG genotype at *NOS3* rs3918181 and AC genotype at rs3918188 was found to be increased in adjusted binary logistic regression analysis, marking the values 2.201, 95%CI 1.167–4.153 (*P* = 0.015) and 2.567, 95%CI 1.317–5.003 (*P* = 0.006) respectively.

### SOD2

Wu et al*.* (2015) reported the association of *SOD2* rs2758331 (A/C) with well-water arsenic, and they found no significant association for this SNP with cardiovascular diseases in Bangladeshi population^28^. But in our study, we found that among iAs-exposed and unexposed patients’ groups, the frequency of AA genotype of *SOD2* rs2758331 was significantly different (13.333, 95%CI 1.280–138.845, *P* = 0.013) (Table 4). Additionally, the genotype distribution difference of this SNP was significant for nail iAs concentration among the patients. The adjusted binary logistic regression analysis showed increased odds of AA genotype in the CVD patients from iAs affected areas (23.674, 95%CI 0.642–872.562), although not significant (*P* = 0.086). The presence of AA genotype increased the odds of cardiac tissue injury significantly (2.490, 95%CI 1.187–5.221, *P* = 0.016). We suspect that AA genotype or A allele of *SOD2* rs2758331 (A/C) might be the risk factors for CVD in iAs exposed patients. Though the finding showed dissimilarities with the previous study and there is not much research on this, therefore, further study should consistently explore the role of *SOD2* in creating oxidative stress and its contribution to CVD in iAs exposed patients.

### APOE

The association of *APOE* with CVD has been reported in various studies^17, 28^. Clark et al*.* (2009) reported that GG genotype of *APOE* rs405509 is significantly associated with hyperlipidemia in Caucasian sample population^55^. Although, in our study, we found no association between *APOE* rs405509 polymorphism and CVD patients from iAs-affected areas (Tables 4 and 5). However, in the adjusted binary logistic regression analysis, the odds of having GT and TT genotypes in the CVD patients from iAs affected areas were higher than the unadjusted analysis, though the OR was not significant. Furthermore, the odds of cardiac tissue injury due to the presence of GT genotypes in this SNP was significantly increased (2.337, 95%CI 1.225–4.459, *P* = 0.010). The results of rs405509 of *APOE* gene of our study thus needs to be validated by involving more CVD patients from iAs affected areas of Bangladesh.

### *ICAM1* and *VCAM1*

Circulating sICAM-1 and sVCAM-1 levels can be used for the prediction of CVD risk^34, 36^. Moreover, these markers are also found to be positively associated with iAs exposure^56^. We found that *ICAM1* rs281432 is significantly associated with iAs exposure and CVD (Table 4). In the earlier studies, the individuals with GG genotype at rs281432 (G/C) of *ICAM1* were found to be affected at a greater extent by cardiovascular effects of iAs exposure in a way that these individuals were genetically predisposed to inflammatory endothelial dysfunction^28, 36^. Similarly, we found a significant difference (12.000, 95%CI 1.325–108.674, *P* = 0.010) in the distribution of GG genotype in iAs-exposed and unexposed patients’ groups (Table 4). The odds of finding GG genotype in CVD patients from iAs affected areas were found to be more increased in adjusted binary logistic regression analysis (18.772, 95%CI 0.858–410.892, *P* = 0.063). The genotype distribution of *ICAM1* rs281432 were also found to be significantly different in case of urinary iAs concentration (*P* = 0.003) among the patients (Table 5). Furthermore, increased odds of cardiac tissue injury was observed among the CVD patients having GG genotype or G allele from iAs affected areas (2.358, 95%CI 1.238–4.493, *P* = 0.009). Thus, findings of our study support the role of endothelial dysfunction as an underlying mechanism of iAs exposure and subsequent adverse cardiovascular effects and allow us to suspect that GG genotype or G allele might be one of the risk factors for CVD among the people living in iAs affected areas.

Although Wu et al*.* (2015) reported a strong association between CC genotype of *VCAM1* rs3176867 with iAs exposure and cardiovascular disease^28^. In our study, we found no significant association with any specific genotypes in iAs-exposed and unexposed patients’ groups (Table 4). However, in this case, the odds of cardiac tissue injury among CVD patients having from iAs affected areas significantly increased with TT genotype at rs3176867 of *VCAM1* (3.520, 95%CI 1.371–9.036, *P* = 0.009) (Table 4). Moreover, the genotypic distribution difference in this SNP was found to be significant in case of urinary iAs concentration (*P* = 0.035) among the patients. Consequently, such discordance between the association analysis results of previous studies and ours need to further investigated.

Additionally, biochemical and haematological profiles of the patients also indicated the associated health complications due to iAs exposure. These parameters do not necessarily confirm or rule out iAs exposure, as various factors other than iAs exposure might also cause their fluctuations. More importantly, these parameters are more difficult to explain in humans where exposure is almost entirely natural and both exposure level and outcomes depend on a combination various factors. However, one noteworthy phenomenon here is that the differences in these parameters were almost always more pronounced in patients from > 50 ppb iAs areas, the higher exposed patient category. Chronic iAs exposure causes damages to internal organs such as the liver, the cardiovascular system, the nervous system, kidneys, and lungs^57^. The significantly higher level of liver function enzymes AST, ALT and serum bilirubin, Kidney function markers serum creatinine, and BUN indicated the arsenic intoxication among the iAs-exposed patients' groups^58, 59^. Arsenic disturbs glucose metabolism by uncoupling of oxidation and phosphorylation, causing excess availability of unutilized glucose^60^. In the study subjects, an increase was found in the RBS levels in the iAs-exposed patients' group. A significant decrease (*P* = 0.003) was found in the levels of serum total protein in the iAs-exposed patients' group. This is because increased breakdown (catabolism) of proteins due to possible oxidative stress by reactive oxygen species generated by arsenic toxicity may contribute to decreased protein levels^60^. Arsenic exposure has been described as a cause of bone marrow depression, causing haematologic abnormalities^61^. This study also reported a significantly lower average white blood cell (WBC) count and platelet count (*P* < 0.05), moderately lower average red blood cell (RBC) count and haemoglobin (Hb) level in the iAs-exposed patients.

**Figure 4:**
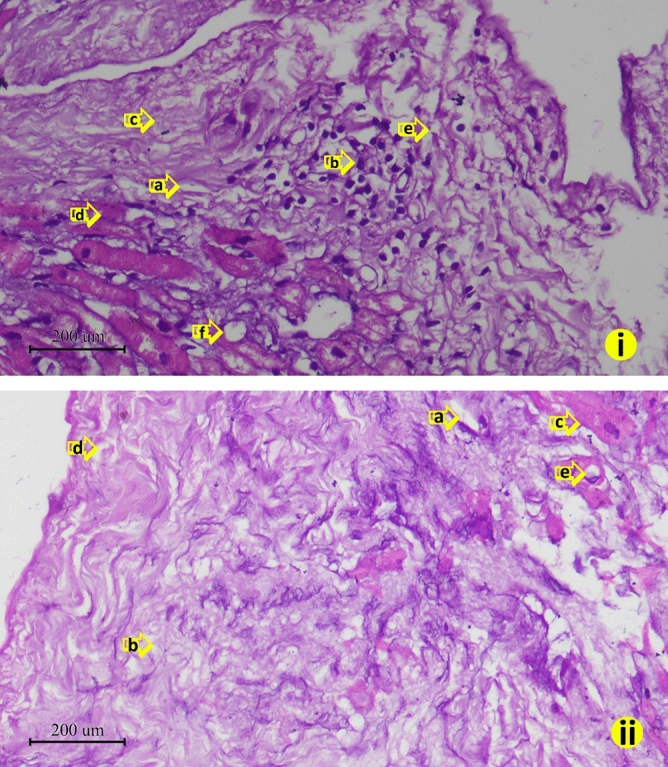
Comparative study of cardiac injury level in between patient from arsenic affected and un-affected area. (i) Patients who came from arsenic affected area showed higher level cardiac tissue injury (**a**) edema; (**b**) leukocyte infiltration; (**c**) fibrosis; (**d**) myocardial fiber swelling; (**e**) fiber separation; (**f**) fatty change than the (ii) patients who came from arsenic un-affected area (**a**) edema; (**b**) leukocyte infiltration; (**c**) fibrosis; (**d**) myocardial fiber swelling; (**e**) fiber separation (H and E stain. Captured in 40x). The default scale bar for each micrograph was set at 200 μm.

In our study, we tried to confirm that iAs-exposure served as one the contributing factors for the CVD among the study subjects and also tried to minimize and exclude other possible contributing factors for the development of CVD among the cardiac surgery patients by involving three criteria. These included, (i) retrieval of National iAs-contamination data of patients’ residential area, (ii) by measuring iAs-concentration of patients’ pre-operative urine and nail; and more importantly, (iii) by measuring iAs-concentration of cardiac tissues collected from the cardiac surgery patients. It may be noted here that confirming iAs-exposure by strictly maintaining these three criteria is relatively difficult than confirming iAs-exposure only by first two of the selected criteria. This is due to the facts that (a) such cardiac tissue collection for research is difficult as patient safety is paramount and (b) cardiac tissue collection from patients who do not meet the first two criteria will go in vain and ultimately jeopardize the actual findings of the study. As such, the number of patients, who meet all the exclusion and inclusion criteria (see Supplementary Method 1) and agree to take part in the research, are relatively low which explains the lower number of subjects involved in this study than the previous studies done by other research groups. However, as in this study, we tried to find an association between iAs exposure and genetic polymorphism CVD patients from different iAs affected areas of Bangladesh, we do believe that it was important to measure the iAs-concentration of cardiac tissues to confirm that the patients most likely developed CVD as a result of cardiac tissue injury caused by iAs-exposure. As all the tissue samples were collected from the CVD surgery patients, tissue injuries were expected to be present in all of them irrespective of arsenic exposure. Arsenic has been reported to induce free radical production and inflammatory activity that might be possible mechanisms underlying iAs exposure and subsequent cardiovascular outcomes^30, 31^. Therefore, increased oedema, leukocyte infiltration, myocardial fibre swelling, fibre separation, fibrosis and fatty changes are the hallmarks of iAs exposure related cardiac injury^62^. In this study, we found higher injury score for these parameters among the iAs-exposed patients (Figs. 3 and 4). In this study, we also observed iAs deposition in the cardiac tissue. However, the comparative study of iAs in cardiovascular tissue is yet difficult due to the lack of "normal values" and iAs speciation in the human tissues, the cardiovascular tissue has been found as one of the good biomarker tissues of the risk to health due to exposure to iAs^62^. iAs-exposed patients have observed to contain a significantly higher concentration of arsenic (*P* < 0.05) in their cardiac tissue samples than the iAs-unexposed patients' group. A strong correlation has also observed between arsenic deposition and genotype. For four genotypes (AA genotype of *AS3MT* rs10748835, AA genotype of *NOS3* rs3918181, GG genotype of *ICAM1* rs281432 and AA genotype of *SOD2* rs2758331), among the seven tested SNPs, were found significantly (*P* < 0.05) associated with the arsenic concentrations in the cardiac tissue. Therefore, this finding strengthens the combined role of iAs exposure and the SNP genotypes in possible association with CVDs.

In conclusion, we propose that the genotypic variants of *AS3MT* rs10748835, *NOS3* rs3918181, *ICAM1* rs281432 and *SOD2* rs2758331 may be associated with CVD risk among the people who live in different iAs affected areas of Bangladesh. We did not find a significant association for rest of the three SNPs of *NOS3*, *APOE* and *VCAM1* genes with the CVD patients from iAs affected areas. In comparison to the reference genotypes of the selected SNPs, AA of *AS3MT* 10748835, AG of *NOS3* rs3918181 and AC of rs3918188, GG of *ICAM1* rs281432, TT of *VCAM1* rs3176867, AA of *SOD2* rs2758331 and GT of *APOE* rs405509 significantly increased odds of cardiac tissue injury of CVD patients from arsenic affected areas. Moreover, the AA genotype of *AS3MT* rs10748835 may be the ultimate susceptibility variant of interest for CVD risk assessment among the people residing in iAs affected areas of Bangladesh. In our future research endeavors, we plan to study the association of the gene polymorphism with arsenic speciation in populations exposed to acute and chronic high iAs levels.

## Materials and methods

### Study subjects

As this was a study involving human subjects, the detail methods, written informed consent form and data collection questionnaire were submitted to the Ethical Review Committee (ERC) of Chittagong Medical College and obtained prior approval. American College of Cardiology guidelines^63^ and additional local guidelines and regulations set by the ERC were followed for safe procedures and collection of patient samples. This was a dual center cross-sectional study involving patients who underwent open-heart surgery at the Department of Cardiac Surgery, Chittagong Medical College Hospital, Chittagong and National Heart Foundation Hospital & Research Institute, Dhaka, and agreed to sign the written informed consent form for participation in this study. Based on the exclusion and inclusion criteria of the study (see Supplementary Method 1), between July 2017 to June 2018, 50 CVD patients were recruited in this study from a total of 270 patients who underwent cardiac surgery at the aforementioned centers making the recruitment percentage of 18.52%. All subjects were asked to fill out a questionnaire, which included queries regarding their lifestyle, area of residence, smoking etc. The questionnaire and the written informed consent form were well explained in the mother language of the patients by a physician of the cardiac surgery team. Patients were divided into two groups after inclusion in this study based on their residential area, whether or not they live in an arsenic contaminated area. The division of patient groups were based on the previously published articles on the ground water arsenic of patients’ residential area^38–45^. After such sorting of patient groups, it was observed that our recruited patients were mostly from the well documented arsenic contaminated areas of Chittagong, Dhaka and Rajshahi divisions of Bangladesh (see Supplementary Dataset).

### Sample collection

Nail (fingers and toes), urine and peripheral blood were collected from each patient shortly after their admission at the aforementioned cardiac surgery centers. Usually the surgery took place within a week of the admission. Nail and urine were used for the iAs exposure measurement of each patient. Peripheral blood was used for biochemical and haematological analysis relevant to this study. A very small portion (0.5 cm × 0.5 cm × 1.0 cm) of the cardiac tissue was collected (see Supplementary Method 2) and was cut in halves and collected in 1 mL phosphate buffer saline (PBS) and 10% neutral buffered formalin, and immediately transported to the laboratory in an ice chest. This cardiac tissue was used for iAs deposition measurement, histopathological and molecular analysis of this study.

### Inorganic arsenic concentration measurements

The collected biological samples of this study (urine, nail and cardiac tissue) were processed for total iAs concentration measurement by Hydride Generation Atomic Absorption Spectrophotometry (AA-7000, SHIMADZU, Kyoto, Japan) as described previously^64^ (for details see Supplementary Methods 3). Background-corrected absorbance values are recorded, and the peak heights are used for quantization using the WizAAard software (SHIMADZU). As arsenobetaine and arsenocholine do not generate the respective hydride under the commonly used analytical condition, therefore the values only indicated the concentration of total iAs^65, 66^. Urinary iAs usually detects iAs exposures that have occurred within the past few days, therefore, used as the main bio-marker of recent exposure. Whereas, nail clippings indicates the integrated exposure to iAs that occurred few months earlier^54^.

### Histopathological analysis of cardiac tissue

For the cardiac tissue histopathological analysis, after collection of the sample by the cardiac surgeons, the atrial part of the tissue were cut and kept separated. Neutral buffered formalin 10% (Sigma-Aldrich, USA) was used as preservative and stored at 4°C until transportation to histopathology lab. At the beginning of tissue processing, the tissue samples were sectioned into small longitudinal and transverse pieces. The small gross sections were put in an automatic tissue processor for dehydration by using gradually increased concentration of ethanol (ranging from 50 to 100% v/v). After dehydration xylene treatment was done sequentially three times to remove the ethanol. Paraffin infiltration was done at 65°C and cavity blocks were used for tissue placement and chilled for hardening. A microtome was used to cut ~ 6 μm thick tissue sections from the paraffin blocks. Sections were placed in a 45°C heated water bath and fixed on a slide after a few minutes. The tissue fixed slides were hydrated by placing in gradually decreasing concentration of ethanol (from 100 to 70% v/v), then stained with hematoxylin and eosin, again rehydrated and washed with xylene and then observed with a light microscope (Olympus, Japan). Oedema, leukocyte infiltration, myocardial fibre swelling, fibre separation, fatty changes and fibrosis were examined for pathological grading of the collected samples. Severity for these parameters were graded with scores from 0 (normal) to 4 (severe). This previously descrived^67^ semi quantitative grading with some modifications showed cardiac injury of a patient as a sum of all parameter scores (for details see Supplementary Methods 4). Average injury score of each parameter was calculated for every patient category.

### Haemato-biochemical assay

Complete blood count (RBC, WBC, Hb and platelet) of the pre-operative blood samples was done by using an automatic haematology analyzer (Beckman Coulter, USA). Other blood serum parameters including creatinine level, total protein, BUN, AST, ALT and RBS by using diagnostic kits manufactured by Human GmbH (Germany) with Erba Chem 5v3 Clinical Chemistry Analyzer (Mannheim, Germany). The details of the assay protocols are described in Supplementary Method 5.

### SNP selection and genotyping

SNPs were selected from the phase III datasets of International HapMap project and further screened with Genome variation Server 138 with the parameters *r*^2^ threshold > 0.8 and allele frequency cutoff ≥ 5% ^68–70^. Most of the selected SNPs were in the intronic region of the genes. However, many of the intronic SNPs fall within the regulatory region (promotors, enhancers etc.) of the gene and can exert effects splicing and expression. Similar importance is put on the role of these cryptic variations in gene regulation. We used Ensembl variant effect predictor to find out if the selected intronic SNPs of this study can affect transcription (see Supplementary Table S7). Results showed SNPs located within regulatory region (*NOS3* rs3918181, *ICAM1* rs281432), non-sense mediated decay variant (*AS3MT* rs10748835, *SOD2* rs2758331, *VCAM1* rs3176867) as well as being located upstream and downstream of the protein coding gene (*NOS3* rs3918188 and *SOD2* rs2758331). All these variations are considered as modifiers with still not known definitive functions.

Genomic DNA from cardiac tissue was isolated by using standard phenol–chloroform-Iso amyl alcohol extraction and ethanol precipitation. SNPs of *AS3MT* rs10748835 and *NOS3* rs3918181 were detected by polymerase chain reaction followed by restriction enzyme digestion with *Apa*LI and *Rsa*I respectively as described previously by Gong and O’Bryant (2012) and Yang et al*.* (2014)^32, 49^. For the rest of selected SNPs, the amplicons were sequenced by capillary electrophoresis sequencing (Macrogen, South Korea). The PCR primers used in this study are given in Supplementary Table S6. Standard PCR amplification for the selected SNPs involved forward and reverse primers (15 pM each), dNTPs (0.2 mM each) (Sigma-Aldrich, Germany), 0.03 MgCl_2_ (Promega, USA), one unit GoTaq Flexi DNA Polymerase (Promega, USA), 1X GoTaq Flexi Reaction Buffer (Promega, USA) and nuclease free water (Invitrogen, USA) in a final reaction volume of 25 μL. The reactions were carried out in a Qantarus Q-Cycler (HAIN Life Science, UK).

### Statistical analyses

All the analyses were done by dividing the patients into two groups: patients from iAs affected areas and patients from iAs unaffected areas. The highest nail iAs concentration of the patients from iAs affected area (276 ppb) was considered as cut off value for the determination of iAs exposed status of the patients; the patients who had higher iAs concentration in nail than 276 ppb was considered iAs exposed, which included all the patients from iAs affected areas. Significant differences were calculated between the two patient groups by Student’s *t* test for age, BMI and iAs concentration in urine, nail and cardiac tissues, and by Chi-square test for sex, smoking habit, hypertension and diabetes mellitus. The differences in the allelic and genotypic frequencies for the selected SNPs were analysed by using Chi-square test between two patient groups. Odds ratios were also calculated in each Chi-square test. Significant differences among the genotypic distribution of the SNPs in regard to iAs concentration in urine, nail and cardiac tissue were calculated between the two patient groups by Student’s *t* test. Single factor ANOVA was done for the analysis of association between biochemical parameters and the SNP genotypes. iAs exposure assessment was done by binary logistic regression between the two patient groups in relation to the iAs concentration measured in urine, nail and cardiac tissue. For toxicokinetic association analysis, binary logistic regression was done among the *AS3MT* rs10748835 genotypes and iAs concentration measured in urine, nail and cardiac tissue between the two patient groups. Furthermore, binary logistic regression was done for the association between all the selected SNP genotypes and total cardiac tissue injury score. All the logistic regression analyses were adjusted for age, sex, BMI, hypertension and diabetes mellitus status of the patients.

## Supplementary Information

Supplementary Information 1.

Supplementary Information 2.

Supplementary Information 3.

Supplementary Information 4.

## Data Availability

All data generated or analysed during this study are included in this article and its Supplementary Table S1-S6, Supplementary Table S7, Supplementary Methods and Supplementary Dataset files.
