# Supplementary material for: Association of arsenic-induced cardiovascular disease susceptibility with genetic polymorphisms
Source: Sci Rep. 2021 Mar 18;11:6263. doi: 10.1038/s41598-021-85780-8 (PMC7973792; doi:10.1038/s41598-021-85780-8)
Supplement: Supplementary file 1 — Supplementary Information 1. [file 41598_2021_85780_MOESM1_ESM.docx]

**Association of arsenic-induced cardiovascular disease susceptibility with genetic polymorphisms**

**Mohammad Al-Forkan^1,*,+^, Fahmida Binta Wali^1,2,+^, Laila Khaleda^1^, Md. Jibran Alam^1^, Rahee Hasan Chowdhury^1^, Amit Datta^1^, Md. Zillur Rahman^3^, Nazmul Hosain^4^, M**ohammad **Fazle Maruf^4^,** Muhammad **Abdul Quaium Chowdhury^4^, NKM Mirazul Hasan^1^, Injamamul Ismail Shawon^1^, Rubhana Raqib^5^**

**^1^Department of Genetic Engineering** and **Biotechnology,** Faculty of Biological Sciences, **University of Chittagong**, Chittagong-4331, Bangladesh.

**^2^University of Science & Technology, Chittagong (USTC)**, Foy’s Lake, Chittagong-4202, Bangladesh.

^3^**Department of Pathology, Chittagong Medical College, Chittagong**-4203, Bangladesh**.** ^4^**Department of Cardiac Surgery, Chittagong Medical College Hospital, Chittagong**-4203, Bangladesh.

**^5^**Infectious Disease Division, International Centre for Diarrhoeal Disease Research, Bangladesh (icddr,b), Mohakhali, Dhaka-1212, Bangladesh.

***Correspondence and request for materials are refered to be addressed to M.A-F (email: alforkangeb@**gmail.com, alforkangeb@cu.ac.bd**)**

^+^These authors contributed equally to this work.

**Supplementary Information**

Supplementary Tables

**Supplementary Table S1:** Allele frequencies of polymorphisms of genes related to arsenic metabolism and their association with CVD.

| Gene | SNP | Allele | Number (%) of patients from iAs-affected areas (n=36) | Number (%) of patients from iAs-unaffected areas (n=14) | OR (95%CI) | *P* value |
| --- | --- | --- | --- | --- | --- | --- |
| **AS3MT** | rs10748835 | A | 28(38.89%) | 11(39.29%) | reference |  |
|  |  | G | 44(61.11%) | 17(60.71%) | 1.017(0.416-2.487) | 0.971 |
| **NOS3** | rs3918181 | G | 20(27.78%) | 17(60.71%) | reference |  |
|  |  | A | 52(72.22%) | 11(39.29%) | **4.018(1.606-10.054)** | **0.002** |
|  | rs3918188 | C | 51(70.83%) | 10(35.71%) | reference |  |
|  |  | A | 21(29.17%) | 18(64.29%) | 0.741(0.294-1.869) | 0.525 |
| **ICAM1** | rs281432 | C | 30(41.67%) | 21(75%) | reference |  |
|  |  | G | 42(58.33%) | 7(25%) | **4.200(1.584-11.138)** | **0.003** |
| **VCAM1** | rs3176867 | C | 40(55.56%) | 15(53.57%) | reference |  |
|  |  | T | 24(33.33) | 7(25%) | 1.286(0.459-3.602) | 0.632 |
|  |  | A | 8(11.11%) | 6(21.43%) | 0.500(0.149-1.683) | 0.258 |
| **SOD2** | rs2758331 | C | 26(36.11%) | 18(64.29%) | reference |  |
|  |  | A | 46(63.89%) | 10(35.71%) | **3.185(1.281-7.915)** | **0.011** |
| **APOE** | rs405509 | G | 30(41.67%) | 16(57.14%) | reference |  |
|  |  | T | 42(58.33%) | 12(42.86%) | 1.867(0.772-4.514) | 0.163 |

The data were analyzed by Chi-square Test and shown as mean ± SE value. Significant values (P<0.05) are typed in bold font.

**Supplementary Table S2.** Association of genetic polymorphisms with patients’ characteristics: Age and BMI.

| **Variable** | **Gene** | **SNPs loci** | **Genotype** | | | **P value** |
| --- | --- | --- | --- | --- | --- | --- |
|  |  |  | **AA** | **Aa** | **aa** |  |
| **Age, y** | ***AS3MT*** | **rs10748835** | 47.54±3.05 | 50.71±1.69 | 48.77±2.18 | 0.656 |
|  | ***NOS3*** | **rs3918181** | 51.05±1.85 | 48.00±2.12 | 47.57±3.03 | 0.496 |
|  |  | **rs3918188** | 52.37±2.44 | 48.27±1.85 | 48.70±2.04 | 0.557 |
|  | ***ICAM1*** | **rs281432** | 50.05±2.23 | 49.18±2.56 | 48.21±2.06 | 0.827 |
|  | ***VCAM1*** | **rs3176867** | 48.65±1.69 | 40.67±4.67 | 51.64±2.06 | 0.120 |
|  | ***SOD2*** | **rs2758331** | 48.88±2.37 | 50.00±2.02 | 48.36±2.64 | 0.877 |
|  | ***APOE*** | **rs405509** | 49.64±2.39 | 49.67±2.13 | 48.33±2.25 | 0.902 |
| **BMI, kg/m^2^** | ***AS3MT*** | **rs10748835** | 22.19±1.12 | 24.98±0.86 | 22.19±0.77 | 0.121 |
|  | ***NOS3*** | **rs3918181** | 23.74±0.73 | 23.90±0.87 | 22.44±1.46 | 0.665 |
|  |  | **rs3918188** | 23.92±1.43 | 24.04±3.87 | 22.98±0.69 | 0.641 |
|  | ***ICAM1*** | **rs281432** | 23.23±0.85 | 24.68±0.83 | 23.45±1.15 | 0.576 |
|  | ***VCAM1*** | **rs3176867** | 23.38±0.73 | 21.36±1.09 | 24.12±0.87 | 0.459 |
|  | ***SOD2*** | **rs2758331** | 23.76±0.73 | 23.49±0.96 | 23.76±0.73 | 0.973 |
|  | ***APOE*** | **rs405509** | 24.08±1.39 | 23.26±0.72 | 23.90±0.92 | 0.799 |

*AS3MT* rs10748835 A/G, *NOS3* rs3918181 A/G, *NOS3* rs3918188 A/C, *ICAM1* rs281432 C/G, *VCAM1* rs3176867 A/C/T (the data for AA genotype was excluded here), *SOD2* rs2758331 A/C and *APOE* rs405509 G/T. *Age and BMI were shown as mean ± SE and analyzed by ANOVA.

**Supplementary Table S3.** Biochemical parameters of CVD patients.

| ***Biochemical***  ***Parameters*** | **Values (Mean± SE)** | | | |  |
| --- | --- | --- | --- | --- | --- |
|  | **Patients from**  **>50 ppb iAs areas (n=27)** | **Patients from**  **>10-50 ppb iAs areas (n=9)** | **Total patients from iAs-affected areas (n=36)*^A^*** | **Patients from**  **iAs-unaffected areas (n=14)** | **P value** |
| ***Serum Creatinine (mg/dl)*** | 1.17±0.04 | 1.08±0.09 | 1.15±0.04 | 0.99±0.03 | **0.026** |
| ***Serum Total Protein (mg/dl)*** | 6.84±0.17 | 7.13±0.29 | 6.92±0.15 | 7.77±0.11 | **0.003** |
| ***Blood Urea Nitrogen (BUN) (mg/dl)*** | 28.99±1.29 | 26.72±2.38 | 28.08±1.14 | 24.43±0.97 | 0.083 |
| ***Serum Bilirubin (mg/dl)*** | 1.19±0.23 | 1.05±0.21 | 1.12±0.18 | 0.98±0.24 | 0.805 |
| ***Alanine Aminotransferase(ALT) (U/L)*** | 40.41±2.45 | 34.56±3.96 | 38.94±2.10 | 30.64±1.76 | **0.033** |
| ***Aspartate Aminotransferase (AST) (U/L)*** | 29.44±1.53 | 25.56±2.59 | 28.14±1.34 | 22.14±1.47 | **0.014** |
| ***Random Blood Glucose (mmol/L)*** | 8.01±0.54 | 7.4±0.46 | 7.86±0.42 | 6.88±0.40 | 0.329 |

***^A^*** Patients from iAs-affected areas (n=36) comprise both Patients from >50 ppb iAs areas (n=27) and Patients from >10-50 ppb iAs areas (n=9).

The data were analyzed by ANOVA and shown as mean ± SE value. Significant values (P<0.05) are in bold font.

**Supplementary Table S4:** Hematological parameters of CVD patients.

| ***Hematological Parameters*** | **Values (Mean± SEM)** | | | |  |
| --- | --- | --- | --- | --- | --- |
|  | **Patients from**  **>50 ppb iAs areas (n=27)** | **Patients from**  **>10-50 ppb iAs areas (n=9)** | **Total patients from iAs-affected areas (n=36)*^A^*** | **Patients from**  **iAs-unaffected areas (n=14)** | **P value** |
| ***Hemoglobin (g/dl)*** | 14.08±0.17 | 14.26±0.41 | 14.12 ±0.16 | 14.58±0.28 | 0.328 |
| ***Total White***  ***Blood Cell count (thous/mm^3^)*** | 8.79±0.20 | 9.02±0.38 | 8.85±0.19 | 9.86±0.19 | **0.010** |
| ***Red Blood***  ***cell count***  ***(million/uL)*** | 4.61±0.08 | 4.62±0.15 | 4.61±0.07 | 4.64±0.24 | 0.989 |
| ***Platelet count (thous/mm^3^)*** | 210.11±13.9 | 259±41.84 | 227.57±17.64 | 313.6±22.97 | **<0.05** |

***^A^*** Patients from iAs-affected areas (n=36) comprise both Patients from >50 ppb iAs areas (n=27) and Patients from >10-50 ppb iAs areas (n=9)

The data were analyzed by ANOVA and shown as mean ± SE value. Significance level, P<0.05.

**Supplementary Table S5:** Parameter-wise average cardiac tissue injury score.

| ***Cardiac Tissue Injury Score(Mean± SE)*** | | | | |
| --- | --- | --- | --- | --- |
| ***Parameters*** | **Patients from >50 ppb iAs areas (n=27)** | **Patients from >10-50 ppb iAs areas (n=9)** | **Total patients from iAs-affected areas (n=36)*^A^*** | **Patients from iAs-unaffected areas (n=14)** |
| ***Oedema*** | 1.22±0.08 | 1.0±0.22 | 1.16±0.8 | 1.0±0.14 |
| ***Leukocyte***  ***Infiltration*** | 0.78±0.26 | 0.2±0.14 | 0.64±0.20 | 0.2±0.11 |
| ***Fibrosis*** | 0.78±0.12 | 0.60±0.17 | 0.72±0.10 | 0.4±0.13 |
| ***Myocardial***  ***Fiber Swelling*** | 1±0.12 | 1±0.0 | 1.0±.09 | 0.8±0.18 |
| ***Fiber***  ***Separation*** | 1.4±0.09 | 1±0.2 | 1.13±0.09 | 0.6±0.13 |
| ***Fatty***  ***Change*** | 1.4±0.09 | 1.22±0.34 | 1.36±0.18 | 0.42±0.22 |
| ***Average cardiac tissue injury score*** | 6.63±0.42 | 4.78±0.56 | 6.17±0.28 | 3.64±0.58 |
|  |  | *P* = 0.001 | |  |

The data were shown as mean ± SE value. Significance level, P<0.05.

***^A^*** Patients from iAs-affected areas (n=36) comprise both Patients from >50 ppb iAs areas (n=27) and Patients from >10-50 ppb iAs areas (n=9). Student’s t-test was performed between the patients’ group of iAs-affected areas and the patients’ group of unaffected areas.

**Supplementary Table S6.** List of PCR primers.

| **SNP** | **Primers** | |
| --- | --- | --- |
|  | **Forward primer** | **Reverse primer** |
| ***AS3MT***  rs10748835 (A/G) | 5’ - CACGTGCAAATGACAACCCCA - 3’ | 5’- GTTTGATTTAGGTTGACTTACA - 3’ |
| ***NOS3***  rs3918181 (A/G) | 5'-CAA CAG TGC AGG TGA ATC TCA -3' | 5'-CAA ACA TTA CCC GCA TCC T -3' |
| ***NOS3***  rs3918188 (A/C) | 5’ - CAAGTGGGGATTCAGCAACTCC - 3’ | 5’- AGCTCGGTGGTCTGGGAACAG - 3’ |
| ***ICAM1***  rs281432 (C/G) | 5’ - GAAGGGTGAGGTTGGCAGAG - 3’ | 5’- GACAAGGACCATAGCCAACTG - 3’ |
| ***VCAM1***  rs3176867 (A/C/T) | 5’ -AGTTTGCCCTCACATCTCG- 3’ | 5’- GTGGTTCTTGTGTGTCAA - 3’ |
| ***SOD2***  rs2758331 (A/C) | 5’- CCTGTGAAGTGAGCCACAGA- 3’ | 5’- ACCCTGCTCATTCTACAGAGA- 3’ |
| ***APOE***  rs405509 (G/T) | 5’- AGCAGGTGCATCATACTGTTCCCAC- 3’ | 5’- CACGAGGTGGGCTGTTCTCCC- 3’ |
