# Supplementary material for: Association of arsenic-induced cardiovascular disease susceptibility with genetic polymorphisms
Source: Sci Rep. 2021 Mar 18;11:6263. doi: 10.1038/s41598-021-85780-8 (PMC7973792; doi:10.1038/s41598-021-85780-8)
Supplement: Supplementary file 3 — Supplementary Information 3. [file 41598_2021_85780_MOESM3_ESM.docx]

**Association of arsenic-induced cardiovascular disease susceptibility with genetic polymorphisms**

**Mohammad Al-Forkan^1,*,+^, Fahmida Binta Wali**^1,2,+^**, Laila Khaleda^1^, Md. Jibran Alam^1^, Rahee Hasan Chowdhury^1^, Amit Datta^1^, Md. Zillur Rahman^3^, Nazmul Hosain^4^, M**ohammad **Fazle Maruf^4^,** Muhammad **Abdul Quaium Chowdhury^4^, NKM Mirazul Hasan^1^, Injamamul Ismail Shawon^1^, Rubhana Raqib^5^**

**^1^Department of Genetic Engineering** and **Biotechnology,** Faculty of Biological Sciences, **University of Chittagong**, Chittagong-4331, Bangladesh.

**^2^University of Science & Technology, Chittagong (USTC)**, Foy’s Lake, Chittagong-4202, Bangladesh.

^3^**Department of Pathology, Chittagong Medical College, Chittagong**-4203, Bangladesh**.** ^4^**Department of Cardiac Surgery, Chittagong Medical College Hospital, Chittagong**-4203, Bangladesh.

**^5^**Infectious Disease Division, International Centre for Diarrhoeal Disease Research, Bangladesh (icddr,b), Mohakhali, Dhaka-1212, Bangladesh.

***Correspondence and request for materials are refered to be addressed to M.A-F (email: alforkangeb@**gmail.com, alforkangeb@cu.ac.bd**)**

^+^These authors contributed equally to this work.

**Supplementary Information**

Supplementary Methods

**Supplementary Method 1:** Study subjects.

The study was a dual center study involving the Department of Cardiac Surgery, Chittagong Medical College Hospital and National Heart Foundation Hospital and Research Institute, Dhaka. The selection of these two centers were approved by the ERC based on their requirement of same level of competence regarding the surgeons, surgery instruments and hospital record facilities. In these two centers, patients from all around Bangladesh come for surgical treatment of cardiac diseases. During the study period (July 2017-June 2018), the patients who were admitted for surgical intervention of diseases, were considered as potential study subjects. The following exclusion and inclusion criteria were followed before recruitment of the patients as study subjects.

Primary exclusion criteria:

(a) Patients aged <20 years and >70 years.

(b) Patients with congenital heart disease.

(c) Patients who expired due to health issues before or after cardiac surgery.

(d) If sufficient quantity of pre-operative nail and urine were not obtained for iAs measurement.

(e) If sufficient cardiac tissue was not possible to obtain for iAs measurement, cardiac histopathology assessment and genomic DNA extraction.

(f) Patients (including guardians of patients) who did not agree to participate.

Secondary exclusion criteria:

(a) If errors had been found in any experimental analyses including iAs measurement (urine, nail and cardiac tissue), cardiac histopathology, haemato and biochemical analyses and SNP analyses; which may be due the sample quality deterioration while transportation or while processing of the samples for analyses.

Inclusion criteria:

(a) Patients aged from 20 to 70 years.

(b) Patients (including guardians of patients) who agreed to participate in the study, signed the informed consent and agreed to the share any required information with the researchers.

(c) If enough samples (nail, urine and cardiac tissue) were obtained for the required analyses.

(d) Patients live in their reported residence permanently.

Primarily 270 hospitalized CVD patients were contacted for inclusion in this study. After selection based on exclusion and inclusion criteria, a total of 50 were recruited in this study.

**Supplementary Method 2:** Sample collection.

(a) Nail sample collection: Nail sample for iAs measurement were collected in polyethylene sterile 15 mL centrifuge tubes. These tubes were prewashed with dilute nitric acid and dried prior to sample collection.

(b) Urine sample collection: Urine samples were collected in sterile polyethylene 50 mL tubes prewashed with dilute nitric acid. Urine samples were mixed with 5-6 drops of 30% nitric acid prior to preservation at -80°C cryofreezer (WiseCryo, South Korea) until further analysis.

(c) Blood sample collection: Pre-operative peripheral venous blood from each patient was collected in two types of blood collection tubes. One was anticoagulant (Potassium-EDTA) containing purple tube and another was clot-activator containing red tube. The collected blood samples were immediately transported to the laboratory for analysis.

(d) Cardiac tissue collection: Cardiac tissue from the patients was collected by taking the opportunity of usual cardiac surgery method which require the heart-lung machine. During the cardiac surgery, to supply the patient with enough oxygenated blood, the patient needed to be put in connection with a heart-lung machine. An incision was made at the right atrium to put the cannula in place to draw the deoxygenated blood from the body. After the surgery, the cannula was removed and the atrium part was sewed in place by the surgeons. The ERC approved by the required safety guidelines, the collection of approximately 0.5cm x 0.5cm x 1.0cm portion from the atrial part of the heart for this study before the reconstruction of the cannula incision. The cardiac tissue sample from each patient was from the same site of right atrium. It was made sure by the surgeons prior to the sample collection that it would not jeopardize the safety and recovery of the patients in any way possible.

**Supplementary Method 3:** Arsenic exposure measurement.

The nail samples were cut into small pieces and pre-washed with non-ionic detergent. Samples were then soaked and washed with deionized water. The washed samples were dried in an oven at 110℃ and kept in a desiccator until analysis is complete.

For urine samples, more than 0.5ml was taken in a 15ml pyrex test tube. The test tube was heated in a dry block just to evaporate the water.

The dried samples were digested with a mixture of concentrated nitric acid and perchloric acid until the solution becomes a water clear. Each digested samples were transferred into a volumetric flask and made up to the mark with distilled water. The digested samples were then pre-reduced to trivalent arsenic (arsenic III) along with a set of standards by adding HCl and KI. These treated samples and standards were introduced into a hydride generation system (HG-AAS, AA-7000, SHIMADZU**,**Kyoto, Japan), where 1M HCl and 0.4% NaBH_4_ were continuously flowing in a reaction coil to generate the gaseous hydride by a peristaltic pump. The arsine gas produced from sodium borohydride and hydrochloric acid were carried into a separator. The separated gas phase was passed on to the absorption cell by argon gas, which was heated by an air-acetylene flame to pyrolyze hydride to arsenic atoms. The atomization step was followed by absorbance measurement and background-corrected (continuous deuterium lamp) absorbance values were recorded. The peak areas were used for quantitation using the WizAAard software (SHIMADZU).

Four working standards (1, 5, 10, 20 ppb) were prepared freshly from an intermediate standard solution (1000 ppb). This intermediate standard were prepared from certified reference material (CRM), Trace CERT, (1000 mg/L As in nitric acid, Sigma-Aldrich Co.) which is traceable to NIST. A standard curve was prepared by the instrument software (WizAAard, SHIMADZU) using the concentration of these known standards and their corresponding absorbance.  Results were automatically calculated by the software based on this standard curve. Samples exceeding the concentration of highest working standard (20 ppb) were diluted and re-analyzed. The arsenic in those samples were determined by multiplying by the appropriate dilution factor.

The method of total inorganic arsenic measurement from heart tissue is similar to above method. These samples are digested with a mixture of concentrated nitric acid and perchloric acid then measured with hydride generation system (HG-AAS, AA-7000, SHIMADZU, Kyoto, Japan).

In house prepared pooled water samples and commercial control material (urine metals toxicology control, UTAK Laboratories Inc, CA 91355) were used to monitor both accuracy and precision.  This pooled sample is stored in aliquots at −20°C and used as an internal quality control in each run/day.  The co-efficient of variation for pooled samples was 2.4% and for CV for control material was 5.8%.  Moreover, known standard samples were also run at regular intervals (every 10-15 run) to check the consistency of instrument response.

**Supplementary Method 4:** Histopathological analysis of cardiac tissue.

For histopathological grading of cardiac tissue injury, the following parameters were chosen: oedema, leukocyte infiltration, fibrosis, myocardial fiber swelling, fiber separation, fatty change, capillary congestion and micro hemorrhage. Based on the presence and severity of these parameters grading was done. An approach described by Gado *et al.* (2013) with some modifications was followed for scoring. Grading for each parameter was performed by using a semi-quantitative scale where 0 was normal and 1-4 represented mild through extensive abnormalities. Total cardiac tissue injury for each patient was calculated as the sum of injury scores of all the parameters for that patient. Average injury score of each parameter was calculated for every patient category.

| **Score** | **Description** |
| --- | --- |
| 0 | No abnormality |
| 1 | Mild injury, affecting <25% of the sample |
| 2 | Moderate injury, affecting 25% - ≤50% of the sample |
| 3 | Severe injury, affecting 50% - ≤75% of the sample |
| 4 | Extensive injury, affecting >75% of the sample |

**Supplementary Method 5:** Haemato-Biochemical Assay.

**Estimation of Biochemical parameters**

The biochemical tests were performed in a reputed diagnostic center. The protocols are described here. Biochemical parameters-

1. Serum Alanine Aminotransferase (ALT),
2. Serum Aspartate Aminotransferase (AST),
3. Blood Urea Nitrogen (BUN),
4. Serum Creatinine,
5. Serum Total Protein,
6. Serum Bilirubin,
7. Random Blood Glucose

**(A) Estimation of Serum Alanine Aminotransferase (ALT)**

**Method:** Kinetic

**Sample:** Serum

**Preparation of Working Reagent:**

To prepare working reagent 2 ml substrate was added with 8 ml buffer (1:4) and mixed.

**Procedure:**

1000 µl of working reagent was taken in a test tube and incubated at 37^o^C for 3-5 minutes. Then 100 µl of sample was added in the same test tube and mixed well. The mixture was placed to the analyzer, and the result was read from the analyzer screen against the blank immediately.

**Assay Conditions:**

Wavelength: Hg 530 nm

Optical Path: 1 cm

Temperature: 37^o^C

**(B) Estimation of Serum Aspartate Aminotransferase (AST)**

**Method:** Kinetic

**Sample:** Serum

**Preparation of Working Reagent:**

To prepare working reagent 2 ml substrate was added with 8 ml buffer (1:4) and mixed.

**Procedure:**

1000 µl of working reagent was taken in a test tube and incubated at 37^o^C for a few minutes. Then 100 µl of sample was added to the same tube and mixed well. Then the mixture was placed to the analyzer, and the result was read from the analyzer screen against the blank immediately.

**Assay Conditions:**

Wavelength: Hg 365 nm

Optical Path: 1 cm

Temperature: 37^o^C

**(C) Estimation of Blood Urea Nitrogen (BUN)**

**Method:** Endpoint

**Sample:** Serum

**Reagent:** Urea-liquicolor

**Procedure:**

| **Pipetted in tubes** | **Reagent Blank** | **Standard** | **Sample** |
| --- | --- | --- | --- |
| R_1_  Distilled water  Standard/R_3_  Sample/Serum | 1 ml  10 µl  ------  ------ | 1 ml  ------  10 µl  ------ | 1 ml  ------  ------  10 µl |

| - Mixed well and kept for 5 minutes at room temperature | | | |
| --- | --- | --- | --- |
| R_2_ | 1 ml | 1 ml | 1 ml |

- Mixed well and kept for 10 minutes at room temperature.
- The absorbances of the sample and the standard were measured against reagent blank at 578 nm from the screen of the analyzer.

**Calculation:**

$$\frac{Absorbance of sample}{Absorbance of standard} \times Conc. of standard (mg/dL)$$

**(D) Estimation of Serum Creatinine**

**Method:** Endpoint

**Sample:** Serum

**Reagent:** Creatinine liquicolor

**Procedure:**

| **Pipetted in tubes** | **Reagent Blank** | **Standard** | **Sample** |
| --- | --- | --- | --- |
| Trichloroacetic acid (TCA)  Distilled water  Standard  Serum | 1 ml  1 ml  ------  ------ | 1 ml  ------  1 ml  ------ | 1 ml  ------  ------  1 ml |

- Mixed well using a glass rod to disperse the precipitate.
- Centrifuged at 3000 rpm for 10 minutes.
- Supernatant was collected in a clean tube.

| **Pipetted in tubes** | **Reagent Blank** | **Standard** | **Sample** |
| --- | --- | --- | --- |
| Picric acid  Alkaline solution  Diluted standard  Supernatant (sample)  Reagent blank (D.W.+TCA) | .5 ml  .5 ml  ------  ------  1 ml | .5 ml  .5 ml  1 ml  ------  ------ | .5 ml  .5 ml  ------  1 ml  ------ |

- Mixed well and kept for 20 minutes at room temperature.
- The absorbances of the sample and the standard were measured against reagent blank at 546 nm from the screen of the analyzer.

**Calculation:**

$$\frac{Absorbance of sample}{Absorbance of standard} \times Conc. of standard (mg/dL)$$

**(E) Estimation of Serum Total Protein**

**Method:** End point

**Sample:** Serum

**Reagent:** Total protein liquicolor

**Procedure:**

| **Pipetted in tubes** | **Reagent Blank** | **Standard** | **Sample** |
| --- | --- | --- | --- |
| Reagent  Standard  Sample | 1 ml  ------  ------ | 1 ml  20 µl  ------ | 1 ml  ------  20 µl |

- Mixed well and kept for 10 minutes at room temperature.
- The absorbances of the sample and the standard were measured against reagent blank at 546 nm from the screen of the analyzer.

**Calculation:**

$$\frac{Absorbance of sample}{Absorbance of standard} \times Conc. of standard (g/dL)$$

**(F) Estimation of Serum Bilirubin**

**Method:** End point.

**Sample:** Serum.

**Reagents:**

1. Reagent (R1):

Sulfanilic acid (3.2mmol/l)

Hydrochloric acid (165mmol/l)

Dimethylsulfoxide (7mmol/l)

1. Reagent (R2):

Sodium nitrite (8.6mmol/l)

**Preparation of working reagent:** Mixing ratio of Rl and R2: 125 ml/25ml

**Procedure:**

| **Pipetted into cuvette** | **Sample** | **Calibrator** |
| --- | --- | --- |
| Working reagent | 1.0 ml | 1.0 ml |
| Sample | 75µl | - |
| Calibrator | - | 75µl |

- Mixed and the absorbance was read against reagent blank after exactly 3 minutes of incubation.

**Calculation:**

$$\frac{Absorbance of sample}{Absorbance of calibrator}\times Conc. of calibrator (mg/dL)$$

**Assay Conditions:**

Wavelength: 555 nm (540-560 nm)

Secondary wavelength: 600 nm

Temperature: 37 °C

Cuvette: 1 cm light path

**(G) Estimation of Random Blood Glucose**

**Method:** Random blood glucose was estimated by Glucose Oxidase method.

**Reagents:** 2N Sodium hydroxide (NaOH), Sodium Sulphate-Zinc sulphate reagent, Phosphate buffer 0.05M pH-7.2, Glucose oxidase reagent.

**Procedure:**

- **Preparation of Test:** 0.1 ml of blood was pipetted into 1.8 ml of sodium sulphate-zinc sulphate reagent in a centrifuge tube. 0.1ml of 2N sodium hydroxide was added and centrifuged at 3000 rpm for 5 minutes. 0.5ml of supernatant was taken in duplicate.
- **Preparation of Blank:** 0.5ml of distilled water was taken.
- **Preparation of Standard:** Standard concentration of glucose was prepared (200mg/dl). 0.5ml of a range of glucose solutions (50mg/dl, 100mg/dl, 150mg/dl and 200mg/dl) suitably diluted from standard were used.

| I. | 50mg/dl – 125µl glucose standard + 375µl distilled water |
| --- | --- |
| II. | 100mg/dl – 250µl glucose standard + 250µl distilled water |
| III. | 150mg/dl – 375µl glucose standard + 125µl distilled water |
| IV. | 200mg/dl – 500µl glucose standard |

- 5ml of the glucose oxidase reagent was added, incubated for 1h at 37°C, and the extinction was read at 540nm against the reagent blank.

**Estimation of hematological parameters**

The hematological tests were performed in a reputed diagnostic center. The protocols are described here. Hematological parameters-

1. Hemoglobin,
2. Total White Blood Cell (WBC) Count,
3. Red Blood Cell (RBC) Count,
4. Platelet Count

**(A) Estimation of Hemoglobin**

Sahli’s method was used for hemoglobin estimation. In this procedure, Sahli’s hemoglobinometer and 0.1 N HCl were used. HCl was taken in a central graduated tube up to mark 2. The blood was sucked in the Sahli’s pipette up to mark 20 (0.02 ml). The blood in the pipette was blown into the graduated tube containing HCl and the pipette was rinsed well by drawing up the acid in the tube several times. The contents of the tube were then stirred well with a glass rod for 3-4 minutes to let the hemoglobin change to acid hematin. The contents of the tube were then diluted with distilled water by adding drop wise, mixed after each addition until it matched the color of the standard comparison tube. The reading from the graduation tube was taken from eye level and from the lower meniscus of the fluid and then noted as mg hemoglobin per 1dL (100 ml) of blood.

**(B) Estimation of Total White Blood Cell (WBC) count**

Total count of White Blood Cell (WBC) was performed by Hemocytometry method. First, the blood was drawn into WBC pipette up to 0.5 mark. Then the WBC diluting fluid was drawn into the WBC pipette upto mark 11. The pipette was rotated between thumb and other fingers horizontally. This gave a dilution of 1:20. The counting chamber of the hemocytometer and the cover slip were cleaned. Then the cover slip was placed in position over the counting chamber by gentle pressure. The fluid in the pipette was then expelled by an angle of 45^o^ and the hemocytometer was allowed to settle down the WBC for 2 minutes. Finally, WBC was counted in the 4 large squares in the corners of the counting chamber (16 small squares).

**Calculation:**

Total WBC count = Cells counted (N)/Volume of all squares × Dilution factor

**(C) Estimation of Red Blood Cell (RBC) count**

Red Blood Cell (RBC) count was performed by Hemocytometry method. In this method, first the blood was drawn into RBC pipette up to 0.5 mark. Then the RBC diluting fluid was drawn into RBC pipette up to mark 101. The pipette was rotated between thumb and other fingers with figure eight movements. This gave a dilution of 1:200. The counting chamber of the hemocytometer and the cover slip were cleaned. Then the cover slip was placed in position over the counting chamber by gentle pressure. A drop of blood was expelled on to the counting chamber by holding the pipette at an angle of 45^o^ and the hemocytometer was allowed for 2-3 minutes to settle down the RBC in the counting chamber.

**Calculation:**

Total RBC count = Cells counted (N)/Volume of all squares × Dilution factor

**(D) Estimation of Platelet count**

In this procedure, 20µl blood was drawn into a WBC pipette. Then, 0.4 ml of platelet diluting fluid (1% ammonium oxalate solution) was drawn immediately into the WBC pipette. This gave a dilution of 1:21. The isotonic balance of the diluent is such that all erythrocytes are lysed while the leukocytes, platelets, and reticulocytes remain intact. The dilution was mixed well and incubated for 20 minutes to allow the lysis of erythrocytes. After that, the dilution was mounted on a hemocytometer. The cells were allowed to settle and then were counted in a specific area of the hemocytometer chamber under the microscope.

**Calculation:**

Total Platelet Count= Cells counted (N)/Volume of all squares × Dilution factor
